# Impact of transcutaneous electrical acupoint stimulation combined with aromatherapy on postoperative nausea and vomiting in patients undergoing microvascular decompression surgery: A prospective randomized study

**DOI:** 10.1016/j.jatmed.2025.02.001

**Published:** 2025-03-27

**Authors:** Yiyuan Luo, Yu Zhang, Qiuyue Fu, Qixing Wu, Tao Zhu, Juan Li, Fang Kang

**Affiliations:** a Department of Anesthesiology, Affiliated Provincial Hospital, Anhui Medical University, Hefei 230001, China; b Department of Anesthesiology, The First Affiliated Hospital of USTC, Division of Life Sciences and Medicine, University of Science and Technology of China, Hefei 230001, China

**Keywords:** Microvascular decompression, Postoperative nausea and vomiting, Transcutaneous electrical acupoint stimulation, Aromatherapy

## Abstract

**Background:**

Microvascular decompression (MVD) surgery is frequently associated with severe postoperative nausea and vomiting (PONV). This prospective randomized trial aimed to evaluate the combined efficacy of transcutaneous electrical acupoint stimulation (TEAS) and aromatherapy on PONV in patients who underwent MVD surgery.

**Methods:**

180 patients who underwent MVD surgery were randomly allocated to each of the four groups: group T (TEAS), group A (aromatherapy), group TA (TEAS combined with aromatherapy), and group C (control). The primary outcome was the incidence of PONV at 24, 48, and 72 h after surgery.

**Results:**

We analyzed the data from 180 patients who completed the trial. During the postoperative 0–24 h, groups T, A, and TA significantly reduced the incidence of nausea compared to group C (31.11 % in group T, 28.89 % in group A, and 24.44 % in group TA *vs.* 60.00 % in group C; *P* < 0.008), and groups A and TA significantly reduced the incidence of vomiting (33.33 % in group A and 22.22 % in group TA *vs.* 62.22 % in group C; *P* < 0.008). During the postoperative 24–48 h, group TA indicated reduced incidence of nausea and vomiting compared to group C (*P* = 0.001, *P* = 0.004, respectively). Furthermore, there were no significant differences in the incidence of nausea and vomiting among four groups within the postoperative 48–72 h (*P* > 0.05).

**Conclusions:**

TEAS and aromatherapy are effective in treating nausea and vomiting following MVD surgery. However, the combined prophylaxis is not superior to either treatment alone.

## Introduction

Postoperative nausea and vomiting (PONV) are one of the most frequent post-neurosurgical complications, causing electrolyte imbalance, intracranial hypertension, and delayed surgical recovery. Furthermore, studies show that PONV prolongs hospital stays, raises healthcare costs, and decreases patient satisfaction with the healthcare system.^1^ Moreover, about 22–70 % of patients who undergo craniotomy suffer from PONV without prophylaxis.^2^ Microvascular decompression (MVD) is a method to treat hemifacial spasms or trigeminal neuralgia that has been associated with an increased risk of PONV, which has an estimated incidence of about 70 %.^3^The increased incidence of PONV might be related to the close operative field proximity to the area postrema (vomiting center) or the chemoreceptor trigger zone.^4^ Currently, combinations of drugs, such as dexamethasone and ondansetron, are recommended for treating PONV.^5^ However, despite antiemetics prophylaxis, the incidence of PONV was still up to 60 % during the first 24 h after craniotomies.^6^ Furthermore, it has been observed that antiemetics have a certain risk of adverse effects, such as extrapyramidal signs, dysphoria, and hypotension.^7^ Nowadays, non-pharmacologic therapy can enhance the quality of postoperative rehabilitation as a complementary therapy since the development of the Enhanced Recovery After Surgery (ERAS) concept. Therefore, it is feasible to explore the use of non-pharmacological interventions besides antiemetic drugs for the management of PONV after craniotomy, including transcutaneous electrical acupoint stimulation (TEAS) and aromatherapy.

TEAS is a non-invasive technique that stimulates surface of the acupoints by low-frequency electrical stimuli. According to Chinese medicine theory, TEAS can activate the meridian qi, allowing blood and qi to flow freely, and possibly aiding in the treatment of vomiting.^8^ Wang et al. ^9^ and Xu et al. ^10^ indicated that TEAS at the PC6 meridian points is a potential adjunct to standard antiemetic treatment for PONV in individuals who underwent supratentorial and infratentorial craniotomy. Lee et al. ^11^ indicated that the stimulation of PC6 acupoint prevented PONV and reduced the requirement for antiemetic rescue. Moreover, stimulation of other acupoints has also been used as a prophylactic treatment for PONV, such as ST36 (Zusanli) and LI4 (Hegu), which also prevent the incidence of nausea and vomiting.^12^ Aromatherapy is another alternative and complementary therapeutic method recommended for reducing the incidence of PONV. Aromatherapy treatment effectively alleviates emotional and physical symptoms through inhalation of vapors or skin absorption of essential oil.^13^ Lavender, lemon, and peppermint are the most commonly utilized essential oils in aromatherapy for topical application (massage or compress) or inhalation for reducing vomiting and nausea.^14^ Rambod et al. ^15^ found that lemon inhalation aromatherapy can reduce PONV, post-surgical retching, and the use of anti-emetic drugs. Another study revealed that inhalation aromatherapy with mint-lemon can improve chemotherapy-induced nausea and vomiting.^16^

However, no prior reports have addressed the role of TEAS and aromatherapy in PONV following MVD surgery. It is still unclear if TEAS, aromatherapy, or a combination of both can successfully decrease the occurrence of PONV in patients who undergoing MVD surgery. Therefore, we conducted this prospective randomized clinical trial to evaluate the effectiveness of TEAS along with lemon inhalation aromatherapy in preventing PONV in these patients.

## Materials and methods

### Study design

This prospective randomized study was approved by the Ethics Review Board of the First Hospital Affiliated of the University of Science and Technology of China (approval number: 2023-ky272) and has been registered in 2023 on www.Chictr.org.cn (registration number: ChiCTR2400079518).

#### Study population

This research included patients who underwent cranial nerve microvascular decompression surgery at the First Hospital Affiliated with the University of Science and Technology of China between December 2023 and June 2024. All the participants were first informed about the trial, and then their consent was acquired before the surgery.

#### Inclusion criteria

Patients who (1) were aged between 18 and 70 years, (2) had ASA class I–II, and (3) signed informed consent after understanding the intervention and evaluations included in the study were included in this study.

#### Exclusion criteria

Patients who (1) had TEAS contraindications such as skin breakage, allergy, or infection, (2) had confirmed sensitivity to plant extracts and olfactory problems, and (3) had confirmed severe psychiatric disorders and epilepsy were excluded from the study.

#### Randomization and allocation

The participants were randomly assigned to four groups: Group T (TEAS), Group A (aromatherapy), Group TA (TEAS combined with aromatherapy), and Group C (control) in a 1:1:1:1 ratio according to computer-generated random sequences. Random sequence numbers were securely enclosed within opaque, sealed envelopes. These envelopes were subsequently revealed by a nurse who was not involved in the patient enrollment or the intervention process. After the allocation sequence was confirmed, the interventions were administered by an anesthesiologist with expertise in acupuncture, who was not involved in any anesthesia administration or data collection activities. Both the outcome assessors and statisticians were blinded to the group assignments. However, due to the distinctive odors used in the aromatherapy (Group A, TA), both the patients and the investigators performing the interventions were likely aware of the group allocations. Although efforts were made to maintain blinding, this sensory exposure could have introduced some degree of unintentional bias.

### Anesthesia and postoperative rescue antiemetic

All patients were induced using the same anesthesia regimen of sufentanil (0.5 μg/kg), propofol (2 mg/kg), rocuronium (0.6–0.9 mg/kg), and dexamethasone (4 mg). To keep the bispectral index (BIS) between 40 and 60 during the surgery, the patient’s anesthesia maintained with sevoflurane (1 %) remifentanil (0.05–2 μg/kg/min), and propofol (4–8 mg/kg/h). If heart rate (HR) > 100 beats/minute, esmolol (10–20 mg) was administered, and at HR < 50 beats/minute, atropine (0.25–0.5 mg) was administered. Furthermore, if the mean blood pressure was < 65 mmHg or reduced by > 20 % from the baseline, 6 mg of ephedrine was administered. Patients who received atropine and ephedrine during surgery were excluded as these drugs influence PONV. Ondansetron (8 mg) and oxycodone (0.1 mg/kg) were administered at dural closure to prevent PONV and hyperalgesia. Moreover, antiemetics (metoclopramide) were administered within 72 h after surgery in the PACU and ward. Subjects who experienced nausea and/or vomiting received antiemetics based on a physician’s decision. Patients received 10 mg IV metoclopramide in the event of severe PONV and their desire for treatment. If the Visual Analog Scale (VAS) score at rest (0 = no pain and 10 = worst pain) was ≥ 4 points or the patients requested analgesia postoperatively, 0.3 g of ibuprofen extended-release tablets were given orally.

### Interventions

In the group T, patients received TEAS by bilateral Hegu (LI4), Neiguan (PC6), and Zusanli (ST36). PC6 is present on the forearm’s volar aspect, 2 cun on the wrist crease, and between the flexor carpi radialis and palmaris longus tendons. LI4 is found at the midpoint of the second metacarpal bone radius on the back of the hand. ST36 is located on the lateral lower leg, 3 cun under the external knee eye. For TEAS, the electrode was attached to the patient’s acupoints on the skin surface and connected with the Huatuo Treatment Instrument (Suzhou Electronic Needle Therapy Instrument SDZ-Ш) via a wire. Then, a distant-dense wave with the frequency of 2/10 Hz was applied. The stimulation intensity was set at the maximum tolerance level. The TEAS was carried out 30 min before anesthesia until the end of the operation and continued for 30 min after extubation in PACU.

In the group A, after the patient recovered from anesthesia and was extubated in the postanesthesia care unit, two drops of lemon essential oil were added to 2 cc distilled water and applied on a 5 × 5-inch gauze that was placed over the patient's mouth and nose, allowing the patients to inhale the evaporated essence for 30 min.^17^, ^18^ This procedure was repeated for 30 min in the morning and afternoon on the first day postoperatively. The lemon essence was a product of Dr. Wong and had 100 % purity. The certificate of decomposition of it was approved and delivered by the quality control unit. According to tests conducted by a professional third-party inspection agency, the major components of lemon essence were limonene, β-terpinene, γ-terpinene, β-caryophyllene, neral α-terpineol, and neryl acetate.^19^

In the group TA, patients in this group received TEAS and lemon essential oil aromatherapy as described in the above groups.

In the group C, the control patients only received standardized perioperative management. Electrodes with attached wires were secured to the specified acupoint locations and linked to the Huatuo Treatment Instrument. However, the stimulator was not powered. Additionally, 2 cc of distilled water was administered to a 5 × 5 inch gauze, which was then placed over the patients' mouths and noses.

### Outcomes

The primary outcome was the incidence of PONV at 24, 48, and 72 h after surgery, including postoperative nausea (PON) and postoperative vomiting (POV). PON is described as an uncomfortable feeling that creates a strong urge to vomit, whereas POV refers to the forceful release of stomach contents through the mouth.

The VAS scores at rest were assessed at 24, 48, and 72 h after surgery, and the total number of patients requiring rescue antiemetics and analgesics was also recorded. Additionally, the surgical and anesthetic data were recorded, as well as the patients' demographic information, which included their PONV risk factors (such as gender, a history of motion, smoking, and PONV).

Adverse events associated with the use of TEAS or lemon essential oil during treatment were also documented. These events included numbness, redness, swelling, skin allergies, and other allergic reactions.

### Statistical analysis and sample size

The sample size was calculated using PASS 15.0 according to the primary endpoint of the PONV incidence. The results of our pilot study showed that the incidence of PONV in the four groups were 0.33, 0.38, 0.23, 0.64 respectively. Therefore, 37 patients in each group were required with an α of 0.05, and β= 0.1. Allowing for a 20 % dropout rate, the sample size was increased to 180 patients (45/group).

We used SPSS 26.0 to assess the data. The normality of the distribution was evaluated through a one-sample Kolmogorov - Smirnov test. Continuous variables were presented as mean ± SD if the data were normally distributed; otherwise, they were presented as median (interquartile range, IQR). The normally-distributed continuous variables were evaluated using an analysis of variance, while the continuous variables that were not normally distributed were assessed through the Kruskal-Wallis test. Categorical variables were presented as numbers (percentages) and analyzed using Pearson’s chi-squared or Fisher's exact tests. All *P-*values of < 0.05 were considered to be statistically significant. The focus of this study was to examine potential differences in the incidence of PONV across the four groups. When a significant *P*-value was indicated (*P* < 0.05), we adjusted for multiple comparisons using Dunn's pairwise test with a Bonferroni correction to control for Type I errors. The Bonferroni adjusted critical *P-*value was calculated as 0.008 (0.05 divided by 6 comparisons). This adjustment was applied to account for the increased risk of false positives due to the multiple statistical tests conducted.

## Results

### Patient characteristics and surgical details

**Fig. 1:**
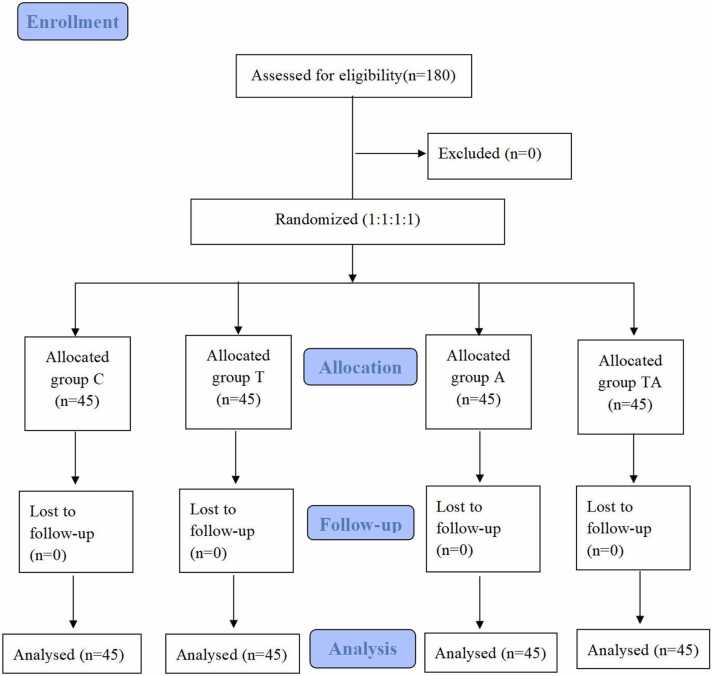
CONSORT flow of clinical procedures for the study.

All the included 180 patients completed the study, and their data were analyzed (Fig. 1). Bradycardia occurred in only two patients during the microvascular decompression procedure for the trigeminal nerve; however, their heart rates normalized after the procedure was paused. Consequently, no patients in this study were excluded due to the administration of atropine or ephedrine. Furthermore, no adverse effects of TEAS or lemon essential oil were observed. The patient’s characteristics, such as gender, motion sickness, age, history of PONV, and history of smoking, were consistent in all the groups. There were no differences in surgical and anesthesia data (*P* > 0.05, Table 1).

**Table 1:** Demographic characteristics of patients, surgery, and anesthesia.

	**Group C (n = 45)**	**Group T (n = 45)**	**Group A (n = 45)**	**Group TA (n = 45)**	*P*-value
Patient					
Gender (M/F)	20/25	16/29	22/23	15/30	0.389
Age (years)	54.69 ± 9.68	52.60 ± 9.60	52.00 ± 7.74	53.09 ± 7.24	0.313
Smoker	9 (20.00)	10 (22.22)	8 (17.78)	9 (20.00)	0.964
Motion sickness	9 (20.00)	15 (33.33)	17 (37.78)	14 (31.11)	0.303
History of PONV	3 (6.67)	3 (6.67)	5 (11.11)	4 (8.89)	0.849
Surgery					
HFS	20 (44.44)	28 (62.22)	27 (60.00)	22 (48.89)	0.261
TN	25 (55.56)	17 (37.78)	18 (40.00)	23 (51.11)	0.261
Duration of surgery (min)	160.73 ± 28.15	150.76 ± 23.22	158.62 ± 26.08	155.56 ± 20.53	0.270
Anesthesia					
Propofol (mg)	680.89 ± 66.63	669.56 ± 62.16	669.33 ± 54.46	668.44 ± 56.37	0.771
Remifentanil (ug)	1930.00 ± 262.94	1871.11 ± 306.65	1820.00 ± 311.59	1885.56 ± 224.02	0.352
Duration of anesthesia (min)	206.78 ± 33.26	200.02 ± 24.54	202.31 ± 24.35	201.00 ± 23.71	0.732

Data are presented as mean ± SD or number of patients (%).

Abbreviation: M: male; F: female; PONV: postoperative nausea and vomiting; HFS: hemifacial spasm; TN: trigeminal neuralgia.

### Comparison of PONV among the four groups

Table 2 presents comparisons of PONV incidence among the four groups. During 0–24 h after surgery, groups T, A, and TA significantly reduced the incidence of nausea compared to group C (31.11 % in group T, 28.89 % in group A, and 24.44 % in group TA *vs.* 60.00 % in group C; *P* < 0.008), and groups A and TA significantly reduced the incidence of vomiting (33.33 % in group A and 22.22 % in group TA *vs.* 62.22 % in group C; *P* < 0.008). However, groups T, A, and group TA did not significantly differ in nausea and vomiting incidence at 0–24 h after surgery (*P* > 0.008). In comparison to group C, the occurrence of nausea and vomiting within 24–48 h post-surgery was significantly lower in group TA (*P* = 0.001, *P* = 0.004, respectively). Conversely, there was no statistically significant decrease in the incidence of nausea and vomiting observed in groups T and A (*P* > 0.008). Moreover, the analysis revealed no statistically significant differences in PONV at 24–48 h after surgery among groups T, A and TA (*P* > 0.008). Furthermore, there were no significant differences in the incidence of nausea and vomiting among four groups within the postoperative 48–72 h (*P* > 0.05).

**Table 2:** Incidence of postoperative nausea and vomiting.

	**Group C (n = 45)**	**Group T (n = 45)**	**Group A (n = 45)**	**Group TA (n = 45)**	*P*-value
Incidence of PON					
0–24 h	27 (60.00)	14 (31.11)*	13 (28.89)*	11 (24.44)*	0.002^#^
24–48 h	21 (46.67)	10 (22.22)	11 (24.44)	6 (13.33)*	0.003^#^
48–72 h	7 (15.56)	4 (8.89)	6 (13.33)	4 (8.89)	0.693
Incidence of POV					
0–24 h	28 (62.22)	16 (35.56)	15 (33.33)*	10 (22.22)*	0.001^#^
24–48 h	23 (51.11)	13 (28.89)	14 (31.11)	10 (22.22)*	0.024^#^
48–72 h	8 (17.78)	4 (8.89)	5 (11.11)	3 (6.67)	0.369

Data are presented as number of patients (%).

^#^*P*-value represents χ2 analysis (<0.05); *significant difference *vs* Group C (<0.008);

Abbreviation: PON: postoperative nausea; POV: postoperative vomiting.

### Comparison of VAS scores at rest among the four groups

**Fig. 2:**
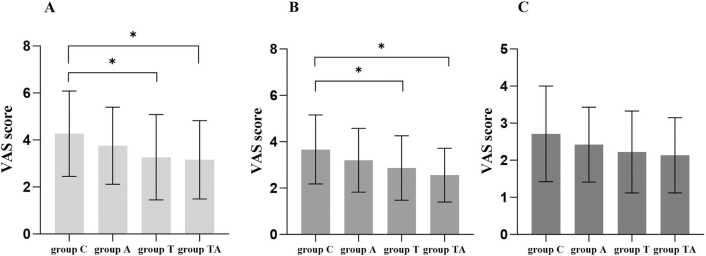
Post-operative VAS scores at rest among the four groups. (A) During the postoperative 0–24 h; (B) During the postoperative 24–48 h; (C) During the postoperative 48–72 h; VAS score: a visual analog scale score; *significant difference vs group C (<0.05).

Fig. 2 shows comparisons of VAS scores among the four groups at 24, 48, and 72 h postoperatively. The VAS scores in group T and TA were significantly lower than those in group C at 24 and 48 h after surgery (*P* = 0.041, *P* = 0.016, *P* = 0.035, *P* = 0.001, respectively).

### Comparison of rescue antiemetics and analgesics among the four groups

Table 3 shows the need for rescue antiemetics and analgesics among four groups within 72 h of surgery. Group TA required fewer rescue antiemetics than group C (*P* = 0.006). Groups TA and T required fewer rescue analgesics than in group C (*P* = 0.002, *P* = 0.003, respectively).

**Table 3:** Need for rescue antiemetics and analgesic.

	**Group C (n = 45)**	**Group T (n = 45)**	**Group A (n = 45)**	**Group TA (n = 45)**	*P*-value
Need for rescue antiemetics					
Metoclopramide	26 (57.8)	15 (33.3)	17 (37.8)	11 (24.4)*	0.027^#^
Need for rescue analgesic					
Ibuprofen	30 (66.7)	16 (35.6)*	20 (44.4)	15 (33.3)*	0.005^#^

Data are presented as number of patients (%).

^#^*P*-value represents χ2 analysis (<0.05); *significant difference vs Group C (<0.008).

## Discussion

The primary finding of this study indicates that the combination of TEAS and aromatherapy is an effective prophylactic measure against PONV following MVD surgery and it reduces the need for rescue antiemetics. However, this combined approach is not superior to either method alone.

PONV is a well-recognized complication that frequently occurs after MVD surgery. Despite the administration of dexamethasone and ondansetron in our control group, the incidence of PONV remained at 60 % within 24 h postoperatively, which is consistent with previous literature.^20^ Our findings also indicated that the incidence of PONV was highest on the first day after surgery and then gradually dropped in the next two days. MVD surgery is frequently associated with PONV because the surgical site is located near the vomiting center in the area postrema, which contains various neuromodulators, including substance P and 5-hydroxytryptamine.^21^ These neuromodulators are closely linked to the onset of nausea and vomiting. Additionally, reduction of the cerebrospinal fluid and spontaneous intracranial hypotension after craniotomy may also result in PONV.^22^ Moreover, any anesthetic medication, proinflammatory stimulus (infection, hypoxia, trauma, etc.), and analgesic drugs can cause postoperative gastrointestinal tract dysfunction, which results in nausea, gas and fluid accumulation, as well as vomiting.^23^ Overall, these results suggest that it is challenging to mitigate the occurrence of PONV in patients undergoing MVD using a single pharmacologic agent.

In our research, TEAS did not reduce vomiting; however, it did decrease the incidence of nausea within 0–24 h following MVD surgery. This finding contradicts the results of a prior study.^24^ The discrepancy may have resulted from differences in acupoint selection and optimal timing. In our study, we selected the bilateral Hegu (LI4), Neiguan (PC6), Zusanli (ST36) acupoints, administering TEAS from 30 min before anesthesia until discharge from the PACU. In contrast, Tu et al. selected bilateral PC6 acupoints and performed acupoint stimulation after the patient regained consciousness in the PACU, with continuous stimulation lasting 30 min. Numerous studies have demonstrated that the application of combined acupoint stimulation is effective in preventing PONV. Alizadeh et al. ^25^ showed that combined stimulation of the PC6 and LI4 acupoints provides significant benefits in preventing PONV. Additionally, the stimulation of acupoints such as PC6, ST36, and ST37 (Shangjuxu) has been shown to effectively alleviate common gastrointestinal complications after surgery, including nausea, vomiting, and abdominal distension.^26^ It has been reported that, compared to acupuncture at a single acupoint, the number of brain regions with altered activity significantly increases following acupuncture at a combination of acupoints.^27^ This finding underscores the synergistic effects associated with the use of acupoint combinations. A previous study describes a potential acupuncture mechanism that modulates the excitability of the parasympathetic and sympathetic autonomic nervous systems and stimulates the secretion of motilin and substance P to exert antiemetic effects.^28^ TEAS can reduce the secretion of 5-HT in the duodenum, suppress activation of the nucleus tractus solitarii in the brainstem, and regulate the levels of neurotransmitters such as serotonin, GABA, and catecholamines.^29^

Lemon essential oil aromatherapy effectively reduced the incidence of PONV within 0–24 h after MVD surgery in our study, which is consistent with the findings of previous research. Hunt et al. ^30^ conducted a randomized trial suggesting that aromatherapy can effectively treat PON. Additionally, Marsh et al. ^31^ reported that an inhaled custom blend of oils, including lemon, can alleviate PONV and reduce the need for antiemetics in orthopedic patients. Aromatic fragrances and oils used in clinical aromatherapy have been shown to effectively improve symptoms such as nausea and vomiting.^32^ Essential oils are fragrant volatile molecules that, when inhaled, stimulate the respiratory, gastrointestinal, and olfactory systems, promoting the release of endorphins to alleviate nausea and vomiting.^33^ Limonene, a primary component of lemon oil, has been demonstrated to reduce the severity of nausea and vomiting induced by serotonergic neurons through the modulation of γ-aminobutyric acid (GABA) secretion.^34^

It was found that both TEAS and aromatherapy were effective in treating nausea and vomiting following MVD surgery. However, this study did not find that the combined intervention was superior to the individual treatments. Notably, the incidence of PONV in the TA group was lower than that in the T and A groups; however, this difference did not reach statistical significance. Consequently, there is no clear indication that the two treatments interact. This may be attributed to a lack of clarity regarding the underlying mechanisms. Although previous research has indicated that the efficacy of either TEAS or aromatherapy in alleviating PONV may be related to pathways associated with serotonin or GABA, the precise mechanisms underlying these effects have yet to be fully elucidated. Further multicenter investigations with larger samples sizes are needed to analyze the potential interactions between these two non-pharmacological therapies.

Our research also revealed that combined prophylaxis and TEAS could significantly reduce VAS scores and the number of patients who need analgesics postoperatively. These results are consistent with previous studies.^35^, ^36^ Acupuncture, a unique therapeutic modality originating from China, demonstrates considerable promise in the clinical management of pain. The analgesic properties of acupuncture are believed to engage multiple molecular and neurotransmission pathways, including 5-hydroxytryptamine, glutamate, and opioid peptides. The literature also has indicated that electroacupuncture and acupuncture can increase the pain threshold, increase the level of endorphins and enkephalins in the body.^37^ Our study found that aromatherapy did no markedly reduce postoperative pain scores, thus differing from other studies.^38^, ^39^ The effects of aromatherapy may vary across essential oil types and surgery types, and it seems that postoperative pain is alleviated more significantly when administered in essential oil form with a higher dosage, concentration, and duration.^40^

This is a single-center study with a relatively small sample size, and there are some limitations. First, the loss of cerebrospinal fluid during the surgery is associated with PONV; however, cerebrospinal fluid leaks are inevitable, and the precise amount of fluid loss is difficult to evaluate. Second, the intensity of acupuncture point stimulation was determined by the patient's subjective feelings, potentially leading to bias in the results. Third, the inhaled dosage of essential oil might be different in every patient, which would affect the outcome. Additionally, two drops of essential oil were used in this research, and the concentration of lemon essential oil might not be enough. Lastly, the present study did not establish distinct blank control groups for each intervention measure. The control group, which consisted of acupoint stimulation without electrical current and gauze saturated with normal saline, may not adequately replicate the placebo effect, potentially biasing the results.

## Conclusions

In summary, TEAS and aromatherapy are effective in treating nausea and vomiting following MVD surgery. However, the combined prophylaxis is not superior to either treatment alone.

## CRediT authorship contribution statement

**Yiyuan Luo:** Writing – original draft, Conceptualization. **Yu Zhang:** Investigation, Data curation. **Qiuyue Fu:** Software, Formal analysis. **Qixing Wu:** Supervision, Methodology. **Tao Zhu:** Validation, Software. **Juan Li:** Writing – review & editing, Supervision. **Fang Kang:** Writing – review & editing, Conceptualization. All authors have read and agreed to the published version of the manuscript.

## Consent for publication

Written informed consent was obtained from all the participants or their legal guardians for their participation in the study.

## Ethical statement

The study was conducted by the Declaration of Helsinki, and approved by the Ethics Committee of the First Hospital Affiliated to the University of Science and Technology of China (approval number: 2023-ky272).

## Funding

This research was funded by by the National Key Research and Development Program of China (STI2030-Major Projects 2021ZD0203100).

## Declaration of competing interest

The authors declare that they have no known competing financial interests or personal relationships that could have appeared to influence the work reported in this paper.

## Data Availability

The datasets used and analyzed during the current study are available from the corresponding author upon reasonable request.
